# Effect of Long-Term Fungicide Applications on Virulence and Diversity of *Colletotrichum* spp. Associated to Olive Anthracnose

**DOI:** 10.3390/plants8090311

**Published:** 2019-08-29

**Authors:** Patrick Materatski, Carla Varanda, Teresa Carvalho, António Bento Dias, Maria Doroteia Campos, Luis Gomes, Tânia Nobre, Fernando Rei, Maria do Rosário Félix

**Affiliations:** 1 ICAAM—Instituto de Ciências Agrárias e Ambientais Mediterrânicas, Instituto de Investigação e Formação Avançada, Universidade de Évora, Polo da Mitra, Ap. 94, 7006-554 Évora, Portugal; 2 INIAV—Instituto Nacional de Investigação Agrária e Veterinária, I. P. Estrada de Gil Vaz, Apartado 6, 7351-901 Elvas, Portugal; 3 Departamento de Engenharia Rural, ICAAM—Instituto de Ciências Agrárias e Ambientais Mediterrânicas, Escola de Ciências e Tecnologia, Universidade de Évora, Polo da Mitra, Ap. 94, 7006-554 Évora, Portugal; 4 Departamento de Fitotecnia, ICAAM—Instituto de Ciências Agrárias e Ambientais Mediterrânicas, Escola de Ciências e Tecnologia, Universidade de Évora, Polo da Mitra, Ap. 94, 7006-554 Évora, Portugal

**Keywords:** anthracnose, control, *Olea europaea* L., fungicides resistance

## Abstract

In this study, the presence and variability of *Colletotrichum* spp. was evaluated by comparing fungal isolates obtained from olive trees under long-time phytosanitary treatments with trees without any phytosanitary treatments (treated and untreated, respectively). Olive fruits of trees of the highly susceptible ‘Galega vulgar’ cultivar growing in the Alentejo region were used as samples. From the 210 olive trees sampled (half from treated and half from untreated orchards), 125 (59.5%) presented *Colletotrichum* spp., with a significant lower number of infected trees in treated (39) when compared to untreated orchards (86). The alignment and analysis of beta-tubulin (tub2), glyceraldehyde-3-phosphate dehydrogenase (GAPDH), actin (ACT), chitin synthase (CHS-1) and histone H3 (HIS-3) gene sequences allowed the identification of all 125 isolates as belonging to the *C. acutatum* complex. The vast majority of the isolates (124) were identified as *C. nymphaeae* and one isolate, from an untreated tree, was identified as *C. godetiae*. Isolates were divided into five different groups: Group A: 39 isolates from treated trees matched in 100% with *C. nymphaeae* sequences from the database; Group B: 76 isolates from untreated trees matched in 100% with *C. nymphaeae* sequences from the database; Group C: one isolate from untreated trees presenting a single nucleotidic difference in the HIS-3 sequence; Group D: eight isolates from untreated trees presenting differences in two nucleotides in the tub2 sequences that changed the protein structure, together with differences in two specific nucleotides of the GAPDH sequences; Group E: one isolate, from untreated olive trees, matched 100% with *C. godetiae* sequences from the database in all genes. Considering the similarities of the sampled areas, our results show that the long-time application of fungicides may have caused a reduction in the number of olive trees infected with *Colletotrichum* spp. but an increase in the number of fruits positive to *Colletotrichum* spp. within each tree, which may suggest different degrees of virulence of *Colletotrichum* isolates from trees growing different management regimes. It is imperative that the fungicides described as causing resistance are applied at appropriate times and intervals, since their efficiency decreases when applied incorrectly and new and more virulent species may arise.

## 1. Introduction

The olive (*Olea europaea* L.) is one of the most cultivated fruit crops in countries characterised by Mediterranean climates and its area is continuously expanding to several countries around the world [1], mainly due to the olive oil health benefits [2]. Portugal is the fourth main olive producer in the European Union; not only does olive has a high economic impact in Portugal, but it also has a significant environmental, social and landscape importance [1]. The construction of the Alqueva dam in the beginning of this century, the biggest water reservoir in Western Europe, has rapidly increased the area of olive orchards in Alentejo region, which is currently responsible for over 70% of the Portuguese olive oil production. Despite the recent increase of super-intensive regime, using mainly ‘Picual’ and ‘Arbequina’ two Spanish cultivars, the Portuguese ‘Galega vulgar’ is still the most cultivated variety, mostly due to the unique characteristics of its olive oil [3]. Olive trees in general and ‘Galega vulgar’ in particular are affected by several diseases. Among them, anthracnose remains the most devastating, capable of destroying entire productions [4,5,6,7]. A large number of species belonging to *Colletotrichum acutatum* and *C. gloeosporioides* complexes are associated to anthracnose in olive [8,9]. Alentejo is located in the southwestern Iberia which is probably the hot spot of olive anthracnose diversity, as it was there that the causal agent was first described [10]. In Alentejo, the disease occurs epidemically and only two species within the *C. acutatum* complex -*C. nymphaeae* and *C. godetiae*- were detected and *C. nymphaeae* highly prevails (>95%) [6]. Despite the fact that, in olive, anthracnose typically affects the fruits near maturation [11], the pathogen can also be present on flowers, leaves, shoots and branches [4,12,13,14]. *Colletotrichum* spp. conidia germinate from acervuli on leaves, twigs and fruits and are dispersed through the rain during the fall when fruits begin to ripen, most of the times becoming the primary inoculum of the disease [4,12,15,16]. Aspects such as environmental conditions (e.g. temperature, rain, humidity), olive cultivar, fruits maturity and integrity and virulence of the pathogen can contribute to the spread and severity of the disease [6,7].

The strategy for anthracnose control is mostly based on fungicide treatments, which are conditioned by environmental conditions, location of the symptoms and disease severity [17]. The fungicides used to control anthracnose can be basically classified according to their ability to move inside the plant. Contact fungicides, such as the ones based on copper, dithiocarbamates and phthalimides act on the surface of leaves or other plant organs mostly by interfering with spore germination or hyphae growth. Penetrant fungicides, such as products based on dicarboximide, cinnamic acid and mandelic acid, move beyond the cuticle of the leaf tissue and have a low translocation within the plant. Lastly, and probably the most used to control olive anthracnose when disease occurs epidemically, the systemic fungicides: they have a large spectrum of mobility through the plant based on the direction and the degree of movement such as xylem-mobile, locally systemic, translaminar and amphimobile, which both protect new host tissue that develops posterior to fungicide application and control established infections [18,19,20,21]. Several systemic fungicides have been used to control *Colletotrichum* spp. such as benzimidazole or benzimidazole fungicide carbendazim (MBC) demethylation inhibitor (DMI), phenylamide, carboximides, strobilurin, hydroxyanilide and carbamate. However, their use on olive has been controlled due to their harmful effect on fruit/oil quality as well as on water and related ecosystems [18,20].

The continuous use of these fungicides over the years has promoted the development of low sensitivity to the fungicides, one of the consequences of the fungicides pressure that selects resistant genotypes, directly decreasing diversity within apathogenic species [22,23], especially due to the high capacity of fast multiplication rates in the resistant genotypes. These pressures trigger mechanisms of variability linked to mutation or sexual reproduction promptly decreasing fungicides efficiency under field conditions [24,25]. This lower sensitivity or resistance to fungicides may, for example, influence the ability to rapidly spread inside the plant reaching a larger number of infected organs, especially because of the disappearance of other isolates sensitive to fungicides, making the disease more severe compared to those wild type isolates that do not present fungicide resistance [22,26]. In fact, some fungicides present modes of action described as gene-specific in terms of inducing resistance. For *Colletotrichum* spp., examples of such fungicides are the products based on strobilurin, that interfere in the mitochondrial respiration pathway by binding to the centre of cytochrome *b* of the pathogenic fungi [27], or products like benzimidazole that work by binding to β-tubulin and thereby prevent the formation of microtubules [28,29,30]. Indeed, β-tubulin is highly related to the emergence of resistance, with several mutation points in the gene of plant pathogenic fungi [31] and particularly in *Colletotrichum* spp. [28].

This study aims to examine the effect of long-time phytosanitary olive treatments on *Colletotrichum* spp. diversity, in terms of five genes, and on *Colletotrichum* spp. virulence, in terms of the number of infected trees and the number of infected olive fruits within each tree. It would be expected that a long-time phytosanitary regime would decrease *Colletotrichum* spp. diversity and increase the capacity of the prevalent genotypes to spread between plants and inside each plant, with higher numbers of infected olive fruits. In this context, it was hypothesised that the orchard management regime (treated or untreated) could contribute to the differences in the *Colletotrichum* spp. genes variability and to differences in the number of infected trees and infected olive fruits within each tree. The following research question was addressed: Does *Colletotrichum* spp. gene variability, the number of *Colletotrichum*-infected olive trees and olive fruits within each tree vary according to the mode of management?

## 2. Results

### 2.1. Colletotrichum spp. Virulence

A total of 125 (59.5%) from the 210 sampled trees were positive to *Colletotrichum* spp. Eighty-six trees (41.0%) belonged to untreated orchards and 39 trees (18.6%) to treated orchards. In the untreated orchards, the number of positive trees to *Colletotrichum* spp. ranged from three to five trees, and in the treated orchards the number of positive trees to *Colletotrichum* spp. ranged from one to four trees (Table 1). Permutational multivariate analysis of variance (PERMANOVA) confirmed the significant higher (*p* = 0.0001) number of trees positive to *Colletotrichum* spp. in untreated olive orchards compared to treated olive orchards (Table 2).

**Figure 1:**
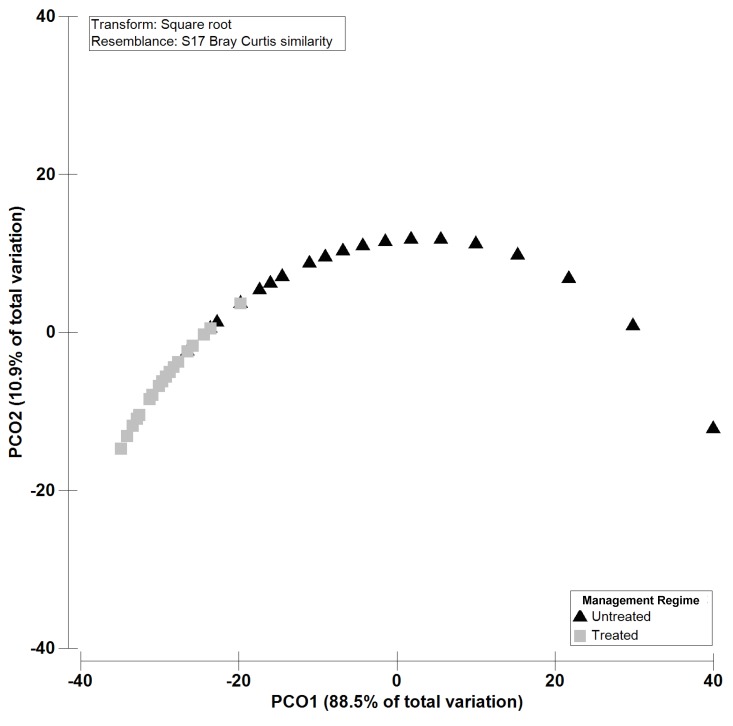
Principal coordinates analysis (PCO) based on the number of fruits showing the presence of *Colletotrichum* spp. dataset for the factor “management regime” (treated and untreated; 1 level, fixed). PCO1 = 88.5% and PCO2 = 10.9%.

In the treated olive orchards, the number of *Colletotrichum* spp.-positive fruits ranged from 18 to 50, and the mean number ± standard error (SE) of fruits positive to *Colletotrichum* spp. per orchard ranged from 5.8 ± 5.8 in plot 3 to 24.4 ± 6.3 in orchard 15 (Table 1). In the untreated orchards the number of fruits positive to *Colletotrichum* spp. ranged from 1 to 26, and the mean number ± SE of fruits positive to *Colletotrichum* spp. per orchard ranged from 1.2 ± 0.4 in orchard 14 and 1.2 ± 0.6 in orchard 21 to 13.6 ± 3.3 in orchard 7 (Table 1). PERMANOVA confirmed the significant higher (*p*= 0.0001) number of olive fruits positive to *Colletotrichum* spp. in the treated compared to the untreated olive trees (Table 2). The Principal coordinates analysis (PCO) ordination performed using the number of *Colletotrichum*-infected fruits clearly reflects a distinct pattern between “Treated and Untreated”, and shows that the first two components (PCO1, 85.1 % and PCO2, 10.9 %) account for 99.4% of the variability of the data (Figure 1).

### 2.2. Isolation, Amplification and Identification of Colletotrichum spp.

Identical isolates within the same tree were considered as a unique isolate. From the 210 olive trees sampled (105 treated and 105 untreated trees), 125 isolates presented morphological and cultural characteristics of *Colletotrichum* spp.; colonies varied from slow to fast growing and from pink to orange colours and conidia of a fusiform shape were observed microscopically. The analysis of ribosomal DNA internal transcribed spacer (ITS rDNA) of all isolates resulted in highly similar sequences to different *Colletotrichum* species, not being able to discriminate among them. The variability of *Colletotrichum* at species level was achieved through the analysis of beta-tubulin (tub2), glyceraldehyde-3-phosphate dehydrogenase (GAPDH), actin (ACT), chitin synthase (CHS-1) and histone H3 (HIS-3) partial genes. The sequence alignment and phylogenetic analysis of the tub2, GAPDH, ACT, CHS-1 and HIS-3 sequences allowed the identification of all 125 isolates as belonging to the *C. acutatum* complex (*C. nymphaeae* and *C. godetiae*). The vast majority of the isolates (124) showed a high similarity to *C. nymphaeae* sequences from the database in the tub2, GAPDH, ACT, CHS-1 and HIS-3 genes, obtained from *Olea europaea* in Portugal and *Anemone* sp. in the Netherlands [32].

In the treated olive trees, all the 39 *Colletotrichum* spp. isolates found matched with a 100% similarity with *C. nymphaeae* sequences from the database in tub2, GAPDH, ACT, CHS-1 and HIS-3 genes (group A) [32]. In the untreated olive trees, 76 (88.4%) matched with a 100% similarity with *C. nymphaeae* sequences from the database in tub2, GAPDH, ACT, CHS-1 and HIS-3 genes (group B) [32]; one isolate (1.2%) revealed a single nucleotide polymorphism (SNP) in the HIS-3 sequences (group C) but showed 100% similarity with *C. nymphaeae* in the remaining genes from the database (tub2, GAPDH, ACT and CHS-1); eight (9.3%) isolates showed differences in the same two nucleotides of the tub2 sequences, together with differences in two specific nucleotides of the GAPDH sequences (group D).

One isolate (1.2%), from untreated olive trees, showed 100% similarity with *C. godetiae* sequences from the database in all genes (group E): tub2, GAPDH, ACT, CHS-1 and HIS-3, obtained from *Olea europaea* in Greece and Italy, respectively, and from *Fragaria* x *ananassa* in Spain [32].

**Figure 2:**
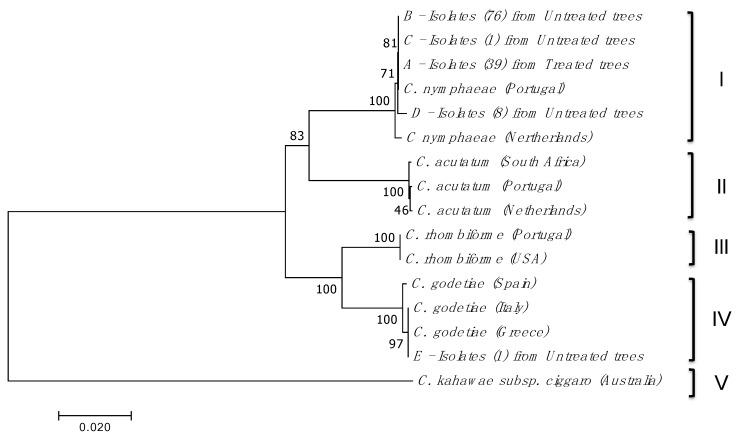
The NJ analysis was constructed from the sequence alignment of tub2, GAPDH, ACT, CHS-1 and HIS-3 (except for *C. gloeosporioides* complex) partial genes from isolates from *C. acutatum* complex (*C. nymphaeae*, *C. godetiae*, *C. acutatum* and *C. rhombiforme*) and *C. gloeosporioides* complex (*C. kahawae* subsp. *ciggaro*), using 125 isolates obtained in this study plus 11 sequences retrieved from the GenBank database, totalling 133 sequences. Repeated sequences within each group of isolates were omitted. Each sequence variant was named with a letter (A to E; except for A and B which are identical but differ in the management regime), following the number of isolates within each group. NJ analysis included 16 sequences. Multiple sequence alignments were generated using MEGA 7 and the neighbour joining (BioNJ algorithms), based on calculations from pairwise nucleotide (nt) sequence distances for gene nt analysis. Numbers above the lines indicate bootstrap scores out of 1000 replicates.

No isolates from either treated or untreated olive trees revealed nucleotide variability in the ACT and CHS-1 genes. The neighbour joining (NJ) analysis was deduced from tub2, GAPDH, ACT, CHS-1 and HIS-3 nucleotide alignments of the isolates (Figure 2). Only one sequence representing each group of isolates, as mentioned above, was used, resulting in: four sequences of *C. nymphaeae* (representing groups A, B, C and D containing 39, 76, 1 and 8 isolates, respectively) and one sequence of *C. godetiae* (representing the group E with a single isolate). The NJ analysis revealed segregation of the isolates into five main clusters (Figure 2). The *C. nymphaeae* cluster (I) appears divided into three subgroups: one subgroup with the Portuguese isolate from the database together with isolates from groups A, B and C, with 39, 76 and 1 isolates, respectively, from treated and untreated olive trees; the other subgroup with isolates from group D with eight isolates from untreated olive trees and the other subgroup with isolate from *Anemone* sp. in the Netherlands retrieved from the database. *C. godetiae* cluster (IV), using the five genes sequences, appears divided into two subgroups: one subgroup with the isolates retrieved from the database from *Olea europaea* in Greece and Italy together with isolates from group E and the other subgroup with isolates from *Fragaria* x *ananassa* in Spain. *C. acutatum* cluster (II), using the five genes sequences, appears divided into two subgroups: one subgroup with the isolates retrieved from the database from *Olea europaea* in South Africa and the other subgroup with isolates retrieved from the database from *Anemone* sp. in the Netherlands and *Olea europaea* in Portugal. *C. rhombiforme* isolates, using the five genes sequences, form the cluster III, with isolates obtained from *Olea europaea* in Portugal and from *Vaccinium macrocarpum* in the USA both retrieved from the database. Cluster V includes *C. kahawae* subsp. *ciggaro* isolates from *Olea europaea* in Australia, using four genes sequences (except HIS-3); this species belongs to *Colletotrichum gloeosporioides* complex.

### 2.3. Gene Sequences Analysis and Characterisation of the Predicted Proteins

**Figure 3:**
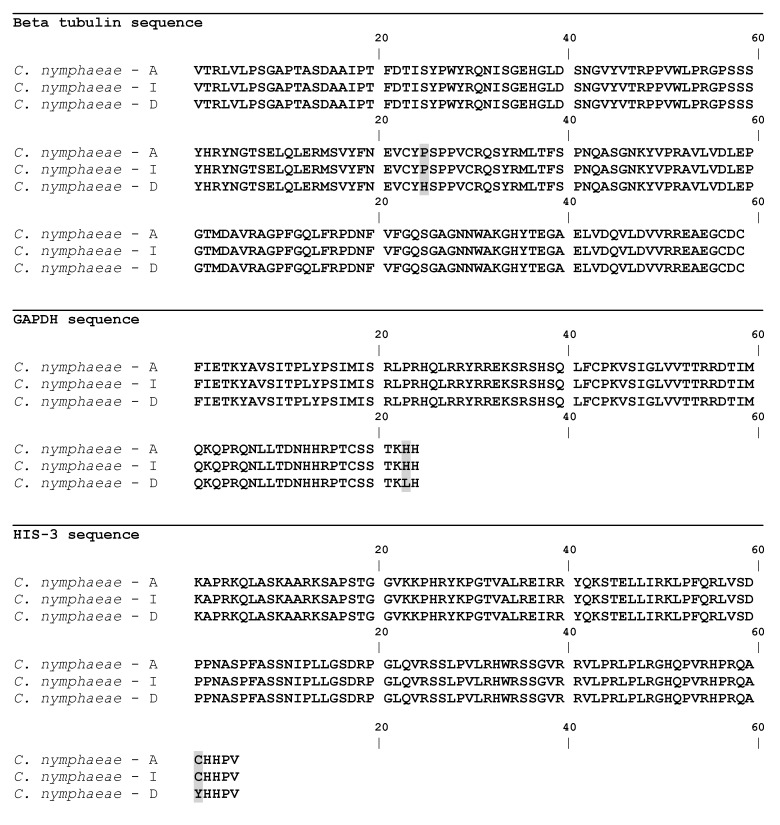
Multiple sequence alignment of the partial Beta tubulin, GAPDH and HIS-3 genes from: *C. nymphaeae*—(*C. nymphaeae* sequences from the database), *C. nymphaeae*—groups A and B, *C. nymphaeae*—group C, and *C. nymphaeae*—group D. Differences in the amino acid are shaded.

**Figure 4:**
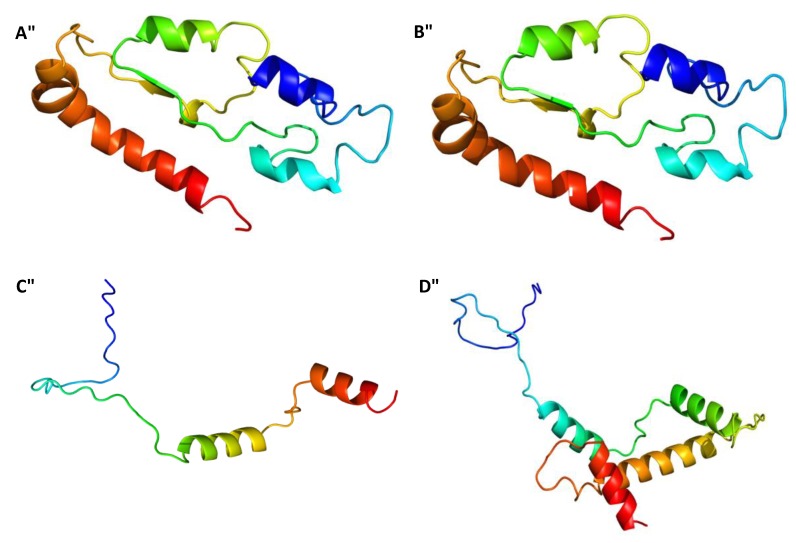
Three-dimensional model predicted for β-tubulin from: A—*C. nymphaeae* isolates from groups A and B and B—*C. nymphaeae* isolates from group D; HIS-3 from: C—*C. nymphaeae* isolates from groups A and B and D—*C. nymphaeae* isolate from group C. The models of beta tubulin were based on a three-dimensional model of the human monomeric kinesin and bovine tubulin as template (Phyre2 fold library ID: c2p4nB) and the models of HIS-3 were based on a three-dimensional model of the “Chicken” (*Gallus gallus*) as template (Phyre2 fold library ID: d1eqzg). The N-terminal is represented in blue and the C-terminal in red.

The β-tubulin multiple sequence alignment of the different isolates with the ones retrieved from the database, allowed to show that only isolates from group D presented differences in the tub2 sequences. From the two nucleotidic differences found, one led to a synonymous change in amino acid 263 of the beta tubulin gene and the other led to a non-synonymous change in amino acid 288 of the tub2 sequence, from a non-polar hydrophobic residue (Proline) to a polar, hydrophobic, aromatic basic and positively charged residue (histidine) (Figure 3). Translation of the tub2 partial complementary DNA (cDNA) revealed a predicted protein with 179 amino acids, a molecular weight of 19.823 kDa and a pI of 5.78. SignalP v. 4.0 allowed detecting an N-terminal peptide of 17 amino acids, with a cleavage site between amino acids 16 and 17 (VRG-QQ), in addition no transmembrane domain was predicted. The protein was confirmed as a member of the Tubulin/FtsZ family, GTPase domain (pfam24077), as determined by Pfam domain search and InterPro scan. This clan contains four families. A three-dimensional model of tub2 (Figure 4) was predicted using the partial β-tubulin of human monomeric kinesin (1bg2) and bovine tubulin (1jff) as template (NCBI TaxId: c2p4nB). There was 81% identity and 110 residues were covered with 100% confidence. The structure showed a single-stranded, right-handed beta-helix fold composed mostly of disordered (30%) followed by Alpha helix (27%) and Beta strand (13%). One beta-strand (four amino acids) was at the N-terminal end of the tub2 followed by one alpha-helix (12 amino acids), one beta-strand (two amino acids) and one alpha-helix (10 amino acids). Another two beta-strands were predicted at the C-terminal end (three and nine amino acids, respectively), followed by one alpha-helix (eight amino acids), one beta-strand (six amino acids) and one alpha-helix (18 amino acids). The tub2 multiple sequences alignment of the different isolates with the ones from the database, showed that isolates from groups A, B and C presented 100% similarity with *C. nymphaeae* sequences from the database (Figure 3). Translation of the tub2 partial cDNA revealed a predicted protein with 179 amino acids and a molecular weight of 19.783 kDa and a pI of 5.59. SignalP v. 4.0 allowed detecting an N-terminal peptide of 17 amino acids, with a cleavage site between amino acids 16 and 17 (VRG-QQ), in addition no transmembrane domain was predicted. The protein was confirmed as a member of the Tubulin/FtsZ family, GTPase domain (pfam24077), as determined by Pfam domain search and InterPro scan. This clan contains four families. A three-dimensional model of tub2 of these isolates was predicted using the partial β-tubulin of human monomeric kinesin (1bg2) and bovine tubulin (1jff) as template (NCBI TaxId: c2p4nB). There was 81% identity and 110 residues were covered with 100% confidence. The structure showed a single-stranded, right-handed beta-helix fold composed mostly of disordered (30%) followed by an Alpha helix (29%) and Beta strand (13%). One beta-strand (four amino acids) was at the N-terminal of the tub2 followed by two alpha-helixes (12 and 15 amino acids, respectively), two beta-strands (five and nine amino acids, respectively) and one alpha-helix (eight amino acids). Another beta-strand was predicted at the C-terminal end (six amino acids) followed by one alpha-helix (17 amino acids) (Figure 4).

The GAPDH multiple sequence alignment of the different isolates with the ones from the database, allowed to show that only isolates from group D presented differences in GAPDH sequences. From the two nucleotidic differences found, one led to a synonymous change in amino acid 33 and the other led to a non-synonymous change in amino acid 254 of the GAPDH gene, from a polar, hydrophobic, aromatic basic and positively charged (histidine) to a non-polar aliphatic and hydrophobic (leucine) (Figure 3). Translation of the GAPDH partial cDNA revealed a predicted protein with 84 amino acids and a predicted molecular weight of 9.956 kDa and a pI of 11.20. SignalP v. 4.0 allowed detecting an N-terminal peptide of 55 amino acids, with a cleavage site between amino acids 54 and 55 (VRG-QQ), in addition no transmembrane domain was predicted. InterPro scan and Pfam domain search did not find any matches to the GAPDH sequences. The three-dimensional model of the GAPDH sequence showed low values of identity (63%) and confidence (38%) and the highest hit obtained by a protein basic local alignment search tool (BLASTp) search was with the human DNA polymerase (PDB Id: 1IH7), for this reason a non-realistic protein model was generated. The GAPDH multiple sequences alignment of the different isolates with the ones from the database, allowed to show that isolates from groups A, B and C matched in 100% with *C. nymphaeae* sequences from the database (Figure 3). Translation of the GAPDH partial cDNA revealed a predicted protein with 84 amino acids and a predicted molecular weight of 9.980 kDa and a pI of 11.20. SignalP v. 4.0 allowed to detect an N-terminal peptide of 57 amino acids, with a cleavage site between amino acids 56 and 57 (VRG-QQ, in addition no transmembrane domain was predicted. InterPro scan and Pfam domain did not find any matches to the GAPDH sequences. The three-dimensional model of the GAPDH sequence showed low values of identity (63%) and confidence (38%) and the highest hit obtained by a BLASTp similarity search was with the human DNA polymerase (PDB Id: 1IH7), for this reason a non-realistic protein model was generated.

The HIS-3 multiple sequence alignment of the different isolates with the ones retrieved from the database, allowed to show that only isolates from group C showed differences in HIS-3 sequences. The unique nucleotidic difference found, led to a non-synonymous change in amino acid 368 of the HIS-3 gene from a polar small tiny sulphur containing (cysteine) to a polar small hydrophobic hydroxylic (tyrosine) (Figure 3). Translation of the HIS-3 partial cDNA revealed a predicted protein with 125 amino acids and a molecular weight of 14.105 kDa and a pI of 12.11. SignalP v. 4.0 allowed to detect an N-terminal peptide of 38 amino acids, with a cleavage site between amino acids 37 and 38 (VRG-QQ), in addition no transmembrane domain was predicted. InterPro scan and Pfam domain search confirmed the protein as a member of the Histone super family, clan histone H2A/H2B/H3/H4 (pfam42403; InterPro IPR000070). This clan contains 17 member families. A three-dimensional model of HIS-3 sequence of this isolate from group C (Figure 4) was predicted using the HIS-3 from “Chicken” (*Gallus gallus*) as template (NCBI TaxId: d1eqzg), which was the top hit species obtained by a BLASTp similarity search. There was a 54% identity and 114 residues were covered with 100% confidence. The structure showed a single-stranded, right-handed alpha-helix fold composed mostly of Alpha helixes (62%) followed by disordered (53%) and Beta strands (0%). One alpha-helix (34 amino acids) was predicted at the N-terminal end of the HIS-3 followed by two alpha-helixes (four and 28 amino acids). At the C-terminal end, another alpha-helix was predicted (11 amino acids). The HIS-3 multiple sequences alignment of the different isolates with the ones from the database, allowed to show that isolates from groups A, B and D matched in 100% with *C. nymphaeae* sequences from the database (Figure 3). Translation of the HIS-3 partial cDNA revealed a predicted protein with 125 amino acids, a molecular weight of 14.045 kDa and a pI of 12.11. SignalP v. 4.0 allowed to detect an N-terminal peptide of 38 amino acids, with a cleavage site between amino acids 37 and 38 (VRG-QQ); in addition, no transmembrane domain was predicted. InterPro scan and Pfam domain search confirmed this protein as a member of the Histone super family, clan histone H2A/H2B/H3/H4 (pfam42403). This clan contains 17 member families. A three-dimensional model of HIS-3 sequence of these isolates (Figure 4) was predicted using the HIS-3 from “Chicken” (*Gallus gallus*) as template (NCBI TaxId: d1eqzg), which was the top hit species obtained by a BLASTp similarity search against the Protein Data Bank. There was 87% identity and 61 residues were covered with 99% confidence. This structure displayed a single-stranded, right-handed alpha-helix fold composed predominantly of alpha helixes (58%) followed by disordered (53%) and Beta strands (0%). Three alpha-helixes were predicted: one at the N-terminal end of the HIS-3 (33 amino acids) followed by another (27 amino acids) and one was predicted at the C-terminal end (11 amino acids).

## 3. Discussion

The disease cycle of anthracnose has been explored for several years [5,33]; however, no study has been conducted in olive to verify the consequences of the continuous use of fungicides in the virulence and diversity of *Colletotrichum* spp. To the best of our knowledge this is the first study focusing on the virulence and diversity of *Colletotrichum* spp. in olive fruits of ‘Galega vulgar’ trees, a cultivar with a high degree of susceptibility to olive anthracnose [14] and considering two opposing management regimes. The study took place in the largest producing olive region in Portugal that is probably the hot spot of olive anthracnose diversity [1] and which significantly contributes to the high European production values [1].

More than 50% of the olive trees sampled showed the presence of *Colletotrichum* spp., a higher value than that found previously in the same region [9,14]. Our findings show a strong and increasing susceptibility of ‘Galega vulgar’ to the anthracnose disease over the years [13,34]. Indeed previous studies have shown that trees from cv. ‘Galega vulgar’ have a higher presence of *Colletotrichum* spp. when compared to other cultivars, such as ‘Cobrançosa’ and ‘Azeiteira’ [14,35]. Additionally to the thinner epidermic cells of the ‘Galega vulgar’ fruits when compared to other cultivars, which are likely to confer a lower protection against pathogens [36]; additionally the higher susceptibility of this cultivar to insect pests such as the olive fruit fly may contribute to the high levels of *Colletotrichum* spp. infestation. The injuries left in the fruit by fly oviposition and/or eclosion can be directly linked to the expansion of the *Colletotrichum* spp. colonization and the consequently to the increase of symptoms [4,37,38].

As expected, treated orchards showed a significant lower number of infected trees (18.6%) when compared to untreated orchards (41.0%); the fungicide treatments tend to limit the number fungal isolates, maintaining resistant ones. The similarities of the sampled areas seem to point out the role of fungicides in the selection pressure in *Colletotrichum* spp., where treated orchards, despite presenting a lower number of infected trees, showed a significant higher number of infected fruits in positive trees, compared to untreated orchards. Therefore, it is plausible to argue that there may exist a possible selective pressure exerted by the application of the fungicides over the years, favouring the evolution and establishment of resistant *Colletotrichum* spp. isolates in the treated olive groves. If the selection pressure is not released, i.e., if fungicide treatments remain under the same regimes in spite of emergence of resistant strains, the spread of resistant isolates is to be expected causing anthracnose to become more devastating over the years, as has been observed in recent studies [14,35]. Hayes et al. [39] suggested that the frequency of fungicide resistance increases with the continued application of fungicides; for example, isolates resistant to strobilurin increased in approximately 70% after one-year application. This urges us to find alternative solutions to fungicide use [40,41,42,43]. A reduction of the application frequencies is one of the most straight forward measures to take, and a good start point until other efficient alternatives come into practice. The use of resistant cultivars can also be an alternative if new groves are being considered. However, this has serious implications in the loss of genetic diversity of important olive cultivars, such as ‘Galega vulgar’ recognised for the high quality of olive oil [3].

The 125 morphologically isolated *Colletotrichum* spp. were confirmed to the genus level by ITS sequence homologies. The lack of genetic diversity of this marker for this genus makes its use unsuitable for species discrimination. The additional partial genes sequences used—tub2, GAPDH, ACT, HIS-3 and CHS-1—allowed species differentiation into the *Colletotrichum* species complex [32]. Previous results in Alentejo region [14], in other Portuguese regions [7,13] and in other Mediterranean countries such as Spain, Italy and Tunisia [5,12,13,44] demonstrated the prevalence of species of *C. acutatum* complex over *C. gloesporioides* complex. This remains valid in the present work. In fact, all these studies verified that the *C. acutatum* complex prevalence in olive has been associated to areas where anthracnose is endemic and more aggressive, as is the case of Alentejo [10]. *C. godetiae* has shown to be present in Alentejo in very low percentages: Materatski et al., [14] showed a presence of 4%, higher than the one found in this study (0.8%) as well as other studies (0%) [9]. Contrasting, *C. godetiae* is the most frequent olive anthracnose pathogen in central Mediterranean countries such as Italy, Montenegro and Greece [5,45], leading to suggest that the species selection is based on the combination of environmental conditions together with the fungicides use. The better adaptation of more virulent species to the environmental conditions of the region, as is the case of *C. nymphaeae* species, associated to possible selection pressure from the intensive use of fungicides may be related to the present results. Nevertheless, the present results confirm that *C. nymphaeae* is the key pathogen in olive anthracnose in Portugal [9,14] as observed in other countries such as Spain, Australia and South Africa [32]. Despite the overwhelming majority of isolates being identified as *C. nymphaeae*, the present study demonstrated that the tub2, GAPDH, ACT, HIS-3 and CHS-1 sequences of the isolates showed a low heterogenicity. However, some variability was observed in isolates from untreated orchards; eight *C. nymphaeae* isolates, showed variability in the tub2 and GAPDH sequences (Group D) when compared with the *C. nymphaeae* isolates from the database [32], the 76 isolates from untreated (Group B); and the 39 isolates (Group A) from treated olive trees. The sequence variability in both genes was in two nucleotides that, in both, led to a synonymous and a non-synonymous change consequently changing one amino acid. GAPDH gene plays a fundamental role in biochemical pathways such as energy metabolism and biomass synthesis [46,47]. Some works have described that mutations in GAPDH gene have an important contribution into the nutrient uptake during the biotrophic phase, as well as a significant functional impact in the expressed enzyme and consequently in the development and pathogenicity of *Colletotrichum* [48,49]. However, no study has demonstrated a direct link between fungicide use and mutations in the GAPDH gene; in fact, due to its high stability, the GAPDH gene is used in fungicide trials for normalization of the gene expression [50]. The confirmation that the differences observed in this gene (eight isolates, group D) have a direct relation to the reduction of the virulence of untreated orchards’ isolates requires additional specific tests. Isolates from group B (*C. nymphaeae* from untreated orchards) did not show any differences in their GAPDH amino acid sequences when compared to isolates from group A (*C. nymphaeae* from treated orchards); in line with the previous hypothesis, it would be interesting to evaluate other fragments of the gene. Contrary to GAPDH, in *Colletotrichum* spp. and in fungal pathogens in general, the β-tubulin gene is known to play a critical role in fungicide resistance and some studies have demonstrated that specific changes in the amino acid positions have been highly indicative of resistance to benzimidazole, methyl benzimidazole carbamate (MBC) and phenylcarbamate fungicides [29,51]. In the present work, a single amino acid replacement was sufficient to modify the protein. Until now there have been no studies of specific changes related to the use of fungicides in the specific amino acid (histidine to proline) of *Colletotrichum* β-tubulin gene; due to its relevance in disease, further research in this area would be highly relevant. Understanding the mechanisms behind the emergence of resistance is a necessary route to its limitation. The eight *C. nymphaeae* isolates from untreated olive trees showed variability in the tub2 predicted protein with the specific increasing of one beta-strand and decreasing of one alpha-helix; changing part of the predicted tub2 protein. These results may indicate a possible binding of fungicides to β-tubulin, although specific studies are needed to demonstrate this.

In line with what happens with the GAPDH gene there are no studies associating resistance to point mutations in the HIS-3 (histone 3), and the differences of one amino acid (cysteine to tyrosine), as well as in the predicted protein of the HIS-3 sequence, found in this work may not be related to fungicides resistance in a specific binding site. In fact, HIS-3, more specifically its tails, are associated to several post-translational modifications such as epigenetic gene regulation [52,53], with activation of gene transcription, and are important for gene silencing [54,55]. The fungicides’ mode of action significantly reduce the capacity of histones to stabilise DNA structure and make chromosomes more vulnerable to breaks [56]; nevertheless, the effect of the fungicides in *Colletotrichum* spp. HIS-3 might be worth further attention.

The majority of *Colletotrichum* isolates (39 from treated and 86 from untreated olive trees) showed no variability in the five genes analysed, although the results have shown different number of fruits positive to *Colletotrichum* spp. within each tree in both treatments, which may suggest different degrees of virulence comparing the two management regimes, although, again, specific competitiveness studies between these isolates are needed to confirm this. Remarkably, until now, studies comparing *Colletotrichum* spp. isolates from large spatial scales in olive anthracnose have given little importance to the variability within the same species, despite many fungicides being described as having a direct mode of action in these genes. In particular, as mentioned above, β-tubulin gene has been widely reported as the gene most associated to fungicides resistance in plant fungal pathogens [28,57,58,59,60]. Therefore, with the exception of tub2, it is likely to suggest that gene mutations associated to the continued use of fungicides may relate to other genes that were not studied here. In addition, it could be possible that other β-tubulin gene mutations may be present in a different region of the gene that was not sequenced. More studies are needed to confirm the direct relation of the fungicides to the prevalence of the existing isolates, namely if prevalent isolates in treated orchards are resistant to the fungicides used. It is imperative that the fungicides described as causing resistance are applied at appropriate times and intervals, since their efficiency decreases when applied incorrectly and new and more virulent species will likely arise [61,62]. Protective applications of fungicides may be effective in controlling resistant *Colletotrichum* isolates and may even contribute to reduce the application of fungicides in general, rationalizing the anthracnose disease management with a direct reduction of the impact on the agroecosystem.

## 4. Conclusions

The majority of *Colletotrichum* spp. isolates sampled in both treated and untreated olive trees belonged to *C. nymphaeae* species, with *C. godetiae* being detected in a unique untreated tree. The present results show that the application of fungicides is likely exerting a selection pressure in *Colletotrichum* spp. towards resistance development, since the olive trees treated with fungicides show divergent results: a lower number of olive trees positive to *Colletotrichum* spp., but a higher number of fruits positive to *Colletotrichum* spp. within each tree, corroborating that fungicide treatments tend to limit possible resistant isolates in a lower number of olive trees. This study suggests that the intensive use of fungicides has likely driven the emergence of resistant *Colletotrichum* spp. isolates in olive trees; however, further studies are needed to confirm the direct relation of prevalent isolates to the resistance to fungicides This shows the importance of developing alternative strategies for the effective and timely management of the olive anthracnose, particularly for the high virulent *Colletotrichum* spp. with possible resistance mutations, in order to change the use of unnecessary fungicide applications that no longer show effect on new resistant *Colletotrichum* spp. isolates. This reduction in selection pressure will restrain the spreading of resistant populations and hopefully revert the situation in the long run.

## 5. Materials and Methods

### 5.1. Study Area and Sample Collection

In the late Spring of 2016, immature olive fruits from ‘Galega vulgar’ cultivar were sampled from 42 orchards randomly selected in three important olive oil-producing sites within the Alentejo region (South of Portugal); Vidigueira, Monforte and Elvas all three regions influenced by the Mediterranean climate. In all sampled orchards the altitude ranged from 156 m to 376 m above sea level, the mean annual temperature ranged from 15.0 °C to 16.5 °C, the annual rainfall ranged from 660 mm to 598 mm, and soils are mostly of schist and calcareous origin. All olive trees sampled were of medium size (with ages ranging from 15 to 20 years) and were planted under a traditional type of management with a spacing of 7 × 7 m. All trees sampled, in both treated and untreated orchards, showed no visible symptoms of anthracnosis. Previous studies on the treated orchards showed a disease level of 35% [14] and no previous studies were performed in the untreated orchards. For the selection of all sampling orchards it was taken into account to choose areas where epidemic outbreaks of anthracnose disease occur on a yearly basis. From the 42 orchards sampled: 21 orchards had been subjected to programmed applications of phytosanitary treatments (treated orchards) and 21 orchards were growing under a conventional mode and had no phytosanitary treatments (untreated orchards). Each sampled orchard consisted in plot of five trees, and at each tree 50 olive fruits were randomly collected around the whole tree at a height of circa 2 m. The olive fruits sampled in both treatments conditions (treated and untreated) presented similar ripening degrees. A total of 10,500 olive fruits (210 trees × 50 fruits at each tree) were sampled from 210 trees (105 treated × 105 untreated). After sampling collection, fruits were stored in the laboratory at 4 °C and processed within the next 48 h. Phytosanitary treatments over the years in the treated olive trees included programmed applications of the fungicides; benzimidazole or MBC, demethylation inhibitor (DMI), strobilurin, copper hydroxide and copper oxychloride, and insecticides; lambda-cyhalothrin, dimethoate and deltamethrin. The applications of the fungicides in the treated olive trees were carried out in different seasons during the olive production cycle, in periods with low probability of rain, so that the product applied would not be washed from the trees, especially the fungicides that act by contact, such those based on copper. Fungicides treatments based on copper (copper hydroxide and copper oxychloride) were done: (i) preventively in the winter (early January) after the olive harvest and tree pruning; (ii) in spring (April/June) where it is already possible to check some anthracnose symptoms; (iii) in autumn (September/October) where the application of these contact fungicides can prevent *Colletotrichum* spore multiplication and spreading. The programmed applications of the systemic fungicides were performed properly due to legislation controlling their application, safeguarding the environment, but in particular safeguarding the chemical residues that may remain in the olive fruit. The applications of the systemic fungicides based on benzimidazole or MBC and demethylation inhibitor (DMI) were carried out: (i) preventively in the winter (early January) after the olive harvest and tree pruning; (ii) and in spring (April/June) at the stage flower buds, period where it is already possible to check some anthracnose symptoms; (iii) in autumn (September/October), when only systemic fungicides based on strobilurin were allowed and they were used to prevent that anthracnose spread epidemically.

### 5.2. Isolation of Colletotrichum spp.

To minimise the epiphytic micro-organisms occasionally adherent to fruit surface, fruit samples were rapidly disinfected with a sequence of 3 minutes immersions in 96% ethanol, 3% sodium hypochlorite solution, 70% ethanol, washed three times in ultra-pure water and dried in sterile paper. After disinfection, fruits were cut lengthwise and placed on 6 cm diameter petri dishes containing potato dextrose agar medium (PDA) (Merck, Darmstadt, Germany) and all plates were then incubated in darkness at 23–25 °C for 5 days. Morphological characteristics—such as the rate of growth, mycelium colour, texture, nature of the growing margin, colour of the reverse side and shape of the conidia observed under an Olympus BX-50 compound microscope (1000× magnification)—were used to select *Colletotrichum* spp. isolates. The whole process was done inside a sterile laminar airflow chamber. The isolates characterised as *Colletotrichum* spp. were transferred to a new (PDA) plate for growing and mycelia was ground in liquid nitrogen and used for DNA extraction for further molecular identification of the species.

### 5.3. DNA Extraction and Identification of Colletotrichum spp.

DNA extraction was performed using the cetyltrimethylammonium ammonium bromide (CTAB) method [63], with some modifications as described previously [64]. DNA concentration was determined by using a Quawell Q9000 micro spectrophotometer (Quawell Technology, Beijing, China). *Colletotrichum* spp. isolates were identified by polymerase chain reaction (PCR) amplification of the internal transcribed spacer (ITS) region (ITS1, 5.8S rRNA, ITS2), partial β-tubulin 2 gene (tub2) (1500-bp), the intron of the Glyceraldehyde-3-phosphate dehydrogenase (GAPDH) (200-bp), partial Actin gene (ACT) (300-bp), partial Chitin synthase (CHS-1) (300-bp) and partial Histone 3 (HIS-3) (402-bp) by using primers: ITS1 and ITS4 [65], T1 and T22 [66], GDFfwd and GDFrev [32,67], ACT-512F and ACT-783R [68] CHS-354R and CHS-79F [68] and CYLH3F and CYLH3R [69], respectively.

PCR reactions were performed in a total volume of 50 μL, containing 30–80 ng of genomic DNA, 10 mM Tris-HCl (pH 8.6), 50 mM KCl, 1.5 mM MgCl_2_, 0.2 mM dNTPs (Fermentas, Thermo Scientific, Waltham, MA, USA), 0.2 μM of each primer and 2.5 U of DreamTaq DNA polymerase (Fermentas, Thermo Scientific, Waltham, MA, USA). The amplification reactions were carried out in a Thermal Cycler (BioRad, Hercules, CA, USA) with an initial temperature of 95 °C for 2 min, followed by 40 cycles of 95 °C for 30 s; 55 °C for 45 s (for ITS), 58 °C for 55 s (for tub2), 56 °C for 55 s (for GAPDH), 62 °C for 40 s (for ACT), 60 °C for 30 s (for CHS-1), 60 °C for 30 s (for HIS-3) and 72 °C for 60 s, as well as a final extension at 72 °C for 10 min.

Amplified products were firstly analysed by electrophoresis in 1% agarose gel and then purified using DNA Clean & Concentrator (Zymo Research, Irvine, CA, USA) according to the instructions of the manufacturer and finally sequenced by Macrogen (Madrid, Spain) in both directions. BLAST, at the National Center for Biotechnology Information (NCBI) was used as a basic local alignment search tool to search for homologous sequences. MEGA software version 7.0 [70] with CLUSTAL W was used to the analysis of the ITS, tub2, GAPDH, ACT, CHS-1 and HIS-3 sequences. Kimura 2-parameter model in MEGA 7 software showed the lowest Bayesian information criterion (BIC) score and was the best-fit nucleotide substitution model for these data. The neighbour joining (NJ) model was used to establish relationships between sequences according to their genetic distances. To evaluate the significance of the interior branches, bootstrap analyses with 1000 replicates were performed. *C. acutatum* (*C. nymphaeae*, *C. godetiae*, *C. acutatum* and *C. rhombiforme*) and *C. gloeosporioides* (*C. kahawae* subsp. *ciggaro*) complexes sequences for the multiple sequence alignments of the five genes (tub2, GAPDH, ACT, CHS-1 and HIS-3) were retrieved from the GenBank (Table 3). An isolate of the *C. gloeosporioides* complex was included and used as outgroup in the sequence alignments. The sequence of gene HIS-3 of the *C. gloeosporioides* (*C. kahawae* subsp. *ciggaro*) complex was not found in database and for this reason was not used in the sequence alignment.

The analyses of the partial proteins were conducted using SignalP 4.0 for prediction of protein signal peptide [71]; ProteParam for determination of the theoretical isoelectric point and protein molecular mass [72]; InterPro scan was performed using the public available software package (https://www.ebi.ac.uk/interpro/sequencesearch/iprscan).

Phyre2 [73] (http://www.sbg.bio.ic.ac.uk/phyre2) was used for the modelling of the tertiary structure of the partial β-tubulin, GAPDH and HIS-3 sequences and the corresponding top hits sequences. Structural classification of the translated partial protein was followed by the convention of SCOPe (Structural Classification of Proteins) (http://scop.berkeley.edu/) [74]. BLASTp search was done against the non-redundant (nr) protein database at NCBI, and sequence selection was based on the TOP hits of Bacteria/Archaea/Fungi/Plants/Insects/others. The percentage of protein identity (identical residues in alignment positions) between the β-tubulin, GAPDH and HIS-3 sequences and those of the other organisms was determined by pairwise comparison.

### 5.4. Statistical Data Analysis

Univariate analyses were performed using data from the two management regimes (treated and untreated) to detect significant differences in the total number of olive trees showing the presence of *Colletotrichum* spp. and in the number of fruits showing the presence of *Colletotrichum* spp. between different olive trees. The statistical analyses were performed using the PRIMER v6 software package [75] with the Permutational multivariate analysis of variance (PERMANOVA) add-on package [76]. The total number of olive trees and the number of fruits positive to *Colletotrichum* spp. were calculated using the dataset from the different management regimes. A one-way permutational analysis of variance (PERMANOVA) was applied to test the hypothesis that significant differences existed in the total number of trees and in the number of fruits positive to *Colletotrichum* spp. among the different management regimes. The PERMANOVA analysis was carried out following the one-factor design: management regime: treated and untreated; 1 level, fixed. Both data were square root transformed in order to scale down the importance of highly abundant replicates and increase the importance of less abundant ones in the analysis of similarity. The PERMANOVA analysis was conducted on a Bray–Curtis similarity matrix [77]. The null hypothesis was rejected at a significance level <0.05 (if the number of permutations was lower than 150, the Monte Carlo permutation p was used). The similarity in the number of fruits within each tree showing the presence of *Colletotrichum* spp. in the different management regimes was plotted by Principal Coordinates Analysis (PCO) using the Bray-Curtis similarity measure based on factor, management regime.

## Figures and Tables

**Table 1:** Mean values ± standard error (SE) (per plot) of infected fruits (50 fruits per tree) and number of infected trees per orchard in Treated and Untreated orchards.

Orchard	Treated		Untreated	
	Infected Trees	Infected Fruits	Infected Trees	Infected Fruits
	Nº of Trees	Mean ± SE	Nº of Trees	Mean ± SE
Orchard 1	1	7.0 ± 7.0	3	2.4 ± 1.4
Orchard 2	1	8.0 ± 8.0	4	3.2 ± 1.7
Orchard 3	1	5.8 ± 5.8	4	2.2 ± 0.7
Orchard 4	2	13.0 ± 8.3	4	1.4 ± 0.7
Orchard 5	2	14.6 ± 9.0	3	2.0 ± 0.9
Orchard 6	1	6.2 ± 6.2	5	3.8 ± 1.2
Orchard 7	1	8.2 ± 8.2	5	13.6 ± 3.3
Orchard 8	2	20.0 ± 12.2	4	9.0 ± 2.8
Orchard 9	2	11.0 ± 7.0	3	5.6 ± 2.8
Orchard 10	2	12.2 ± 7.5	5	9.8 ± 1.9
Orchard 11	2	10.0 ± 6.2	5	3.0 ± 1.1
Orchard 12	2	18.6 ± 11.4	5	2.6 ± 0.5
Orchard 13	2	12.4 ± 7.8	5	8.6 ± 4.1
Orchard 14	2	8.0 ± 4.9	4	1.2 ± 0.4
Orchard 15	4	24.4 ± 6.3	5	6.4 ± 3.5
Orchard 16	2	11.6 ± 7.1	3	4.2 ± 2.9
Orchard 17	2	11.0 ± 7.0	5	12.6 ± 2.7
Orchard 18	2	19.2 ± 11.8	3	1.4 ± 0.7
Orchard 19	2	10.4 ± 6.4	3	3.4 ± 1.4
Orchard 20	2	14.6 ± 9.0	5	8.2 ± 2.1
Orchard 21	2	14.6 ± 9.1	3	1.2 ± 0.6

**Table 2:** Details of the one-factor PERMANOVA dataset test on the number of fruits and number of trees positive to *Colletotrichum* spp. for the factor; management regime “Treated and Untreated” (1 level, fixed) for the variables analysed.

Samples	Source	Degrees of Freedom	Sum of Squares	Mean Squares	Pseudo-F	Unique Perms	p (Perm)
Infected fruits	Treated *versus* Untreated	1	51773	51773	139.17	9942	**0.0001**
	Residual	123	45757	372.01			
	Total	124	97530				
Infected trees	Treated *versus* Untreated	1	4167	4167	77.073	638	**0.0001**
	Residual	40	2162.6	54.065			
	Total	41	6329.6				

**Table 3:** Strains of *Colletotrichum* spp. used in the sequence alignments, with collection details and GenBank accession numbers.

Species	Host	Country	GenBank Accession Number				
			TUB2	GAPDH	ACT	CHS-1	HIS-3
*C. nymphaeae*	*Olea europaea*	Portugal	JQ949852	JQ948531	JQ949522	JQ948862	JQ949192
*C. nymphaeae*	*Anemone* sp.	The Netherlands	JQ949876	JQ948555	JQ949546	JQ948886	JQ949216
*C. godetiae*	*Olea europaea*	Greece	JQ950066	JQ948746	JQ949736	JQ949076	JQ949406
*C. godetiae*	*Olea europaea*	Italy	JQ950064	JQ948744	JQ949734	JQ949074	JQ949404
*C. godetiae*	*Fragaria x ananassa*	Spain	JQ950075	JQ948755	JQ949745	JQ949085	JQ949415
*C. acutatum*	*Olea europaea*	South Africa	JQ950014	JQ948694	JQ949684	JQ949024	JQ949354
*C. acutatum*	*Olea europaea*	Portugal	JQ950015	JQ948695	JQ949685	JQ949025	JQ949355
*C. acutatum*	*Anemone* sp.	The Netherlands	JQ950017	JQ948697	JQ949687	JQ949027	JQ949357
*C. rhombiforme*	*Olea europaea*	Portugal	JQ950108	JQ948788	JQ949778	JQ949118	JQ949448
*C. rhombiforme*	*V. macrocarpum*	USA	JQ950109	JQ948789	JQ949779	JQ949119	JQ949449
*C. kahawae* subsp. *ciggaro*	*Olea europaea*	Australia	JX010434	JX009966	JX009523	JX009800	-
